# Dissecting Dynamic Genetic Variation That Controls Temporal Gene Response in Yeast

**DOI:** 10.1371/journal.pcbi.1003984

**Published:** 2014-12-04

**Authors:** Avital Brodt, Maya Botzman, Eyal David, Irit Gat-Viks

**Affiliations:** Department of Cell Research and Immunology, Tel Aviv University, Tel Aviv, Israel; University of Cologne, Germany

## Abstract

Inter-individual variation in regulatory circuits controlling gene expression is a powerful source of functional information. The study of associations among genetic variants and gene expression provides important insights about cell circuitry but cannot specify whether and when potential variants dynamically alter their genetic effect during the course of response. Here we develop a computational procedure that captures temporal changes in genetic effects, and apply it to analyze transcription during inhibition of the TOR signaling pathway in segregating yeast cells. We found a high-order coordination of gene modules: sets of genes co-associated with the same genetic variant and sharing a common temporal genetic effect pattern. The temporal genetic effects of some modules represented a single state-transitioning pattern; for example, at 10–30 minutes following stimulation, genetic effects in the phosphate utilization module attained a characteristic transition to a new steady state. In contrast, another module showed an impulse pattern of genetic effects; for example, in the poor nitrogen sources utilization module, a spike up of a genetic effect at 10–20 minutes following stimulation reflected inter-individual variation in the timing (rather than magnitude) of response. Our analysis suggests that the same mechanism typically leads to both inter-individual variation and the temporal genetic effect pattern in a module. Our methodology provides a quantitative genetic approach to studying the molecular mechanisms that shape dynamic changes in transcriptional responses.

## Author Summary

Genetic variation is postulated to play a major role in transcriptional responses to stimulation. Such process involves two inter-related dynamic processes: first, the time-dependent changes in gene expression, and second, the time-dependent changes in genetic effects. Although the dynamics of gene expression has been extensively investigated, the dynamics of genetic effects yet remain poorly understood. Here we develop DyVER, a method that combines genotyping with time-series gene expression data to uncover the timing of transitions in the magnitude of genetic effects. We examine gene expression in yeast segregants during rapamycin response, finding several distinct ways of change in the magnitude of genetic effects over time. These include impulse-like and sustained transitions in genetic effects, acting both in *cis* and *trans*. Our findings suggest that associations of genes with the same genetic variant often occur via the same timing of state transition in genetic effects. Furthermore, the results uncover a previously unknown variant whose impulse-like temporal genetic effect suggests a novel molecular function for determining the timing rather than the magnitude of response. Our results show that steady-state association studies miss important genetic information, and demonstrate the power of DyVER to render a comprehensive map of dynamic changes in genetic effects.

## Introduction

**Figure 1:**
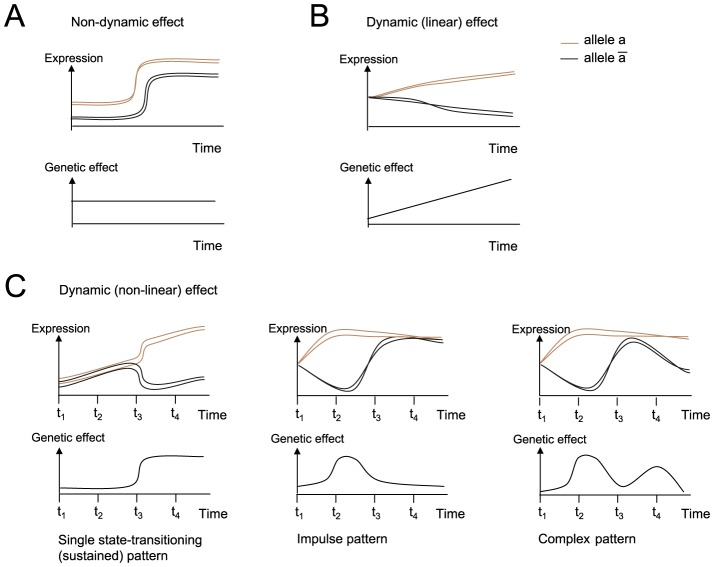
Temporal genetic effect patterns. Schematic view of gene expression patterns (top) and the relevant temporal genetic effects for these genes (bottom). The cartoons demonstrate a *non-dynamic genetic effect pattern* (**A**), a *dynamic, linear genetic effect pattern* (**B**), and a *dynamic*, *non-linear genetic effect pattern* (**C**). Top: shown are gene expression levels (*y*-axis) during a response to stimulation (*x-*axis). Each curve represents measurements in a different homozygous animal strain (segregants), where brown or black indicates whether the genotype of the associated genetic variant is 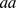 or 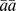, respectively, in each strain. Bottom: shown are *genetic effects* (that is, the change in gene expression between the 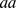 -carrying and 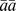 -carrying strains, *y*-axis) during a response to stimulation (*x-*axis). (**C**) Examples of non-linear genetic effect patterns, which are the focus of this study, including (left to right) a *single state-transitioning* pattern, which may be followed by a sustained new level of genetic effect, a *single-pulse* (*impulse*) pattern, and a multiple-pulse (*complex*) genetic effect pattern.

Inherited variation in gene expression is likely to have a major effect on cellular and disease phenotypes, and may allow the underlying DNA polymorphisms (genetic variants) to be identified [1]. The *genetic effect* of a particular variant on a certain RNA is the quantitative change in gene expression that is associated with changing the variant's genotype (allele). Two recent studies have demonstrated that genetic effects on longitudinal gene expression data might be either stable – where the genetic effect is similar at all time points (a *non-dynamic effect pattern*; **Fig. 1A**) – or flexible, changing the magnitude of effect during time points (*a dynamic effect pattern*; **Fig. 1B,C**) [2], [3].

Dynamic effect patterns may be described in terms of the shape of changes in genetic effects over time. A *linear-like genetic effect pattern* (**Fig. 1B**) reflects a gradual change in the magnitude of genetic effects, whereas in a *non-linear genetic effect pattern* (**Fig. 1C**), the level of genetic effect is sustained in some time periods and spikes up or down in others (**Fig. 1C**). In most studies, transcription responses across individuals have been monitored only in two time points (before and after stimulation) and therefore the dynamics of changes in genetic effects over time could not be characterized [4]–[9].

Understanding non-linear genetic effects can, in principle, allow the *timing* of influence of certain regulatory mechanisms to be revealed. For example, a single state-transitioning in genetic effects may uncover the timing of alteration in a regulatory mechanism interacting with a genetic variant (e.g., transition to a new steady state at t_3_, **Fig. 1C**, **left**). Such a mechanism can be revealed even when additional mechanisms are acting in parallel (e.g., up-regulation during the entire time course; **Fig. 1C**, **left**). The linear genetic effect pattern, in contrast, lacks sharp alterations and therefore does not specify finely-timed information about regulatory mechanisms (**Fig. 1B**).

This study is focused on mapping temporal patterns of non-linear genetic effects and using this information to address major questions about dynamic transcription responses. Which dynamic genetic effect patterns are prevalent in global gene responses? Are there any general principles - either functional or mechanistic - shared among genes carrying the same temporal genetic effect patterns? Can we derive insights about the mechanisms underlying such dynamic genetic effect patterns?

Here we developed DyVER (Dynamic Variant Effect on Response), a statistical framework to predict genetic variants and study their dynamic changes in genetic effect sizes. DyVER was mainly designed to achieve an accurate detection of non-linear genetic effects (**Fig. 1C**) during time points. The methodology is based on the notion of a two-state digital model that pinpoints the particular time point at which a rapid change in genetic effects occurs; it is therefore suitable for revealing the timing of state transitions in genetic effects. DyVER takes as input synchronous data in several time points and across a population, and is tailored for recombinant inbred strains that are commonly utilized in genetic studies [2], [10]–[14].

DyVER differs from extant genetic approaches in several aspects. First, some existing methods construct a full model of the response curve across individuals. Their number of parameters is therefore increasing with the number of time points (*e.g*., [15]). DyVER, in contrast, is primarily designed for the specific task of identifying the time points of alterations in effect sizes. This partial modeling allows the use of only a small number of parameters regardless the number of time points and the shape of the temporal pattern. Secondly, DyVER is focused on modeling the dynamics in genetic effects while eliminating the confounding gene expression variables. This is unlike extant approaches, which commonly fit both gene expression and genetic effects to a certain function over time [11], [15]–[20]. Finally, if desired, DyVER can exploit the order in the input time course data, unlike several approaches that are based on unordered correlated traits (e.g., multivariate methods [21], [22] or dimension reduction methods [23]). Notably, DyVER is a practical translation of differential expression approaches (with or without time-series data [24]–[26]) for the case of statistical genetic studies.

Here we report on the use of DyVER to investigate temporal gene responses at six time points after stimulation with the TOR inhibitor rapamycin and across genotyped yeast segregants [27]. The results depict a complex map of non-linear changes in genetic effects. We identify a causal variant that affects the timing of spike up in transcript levels. Importantly, our findings suggest a previously unknown high-order temporal coordination of genetic effects: modules of genes influenced by a common dynamic genetic variant not only participated in the same biological pathway, but also shared orchestrated dynamics of genetic effects. Based on this modularity, we hypothesize that in some cases dynamic effect patterns are a property of the regulatory mechanism within which a genetic variant resides (rather than a property of the target responding transcript). We demonstrate that using this notion it is possible to enhance the identification of underlying causal genes based on their characteristic temporal effect pattern. Our results indicate the utility of studying dynamic genetic effects acting on global gene transcription.

## Results

### DyVER: A method for inferring dynamic, non-linear genetic effects

**Figure 2:**
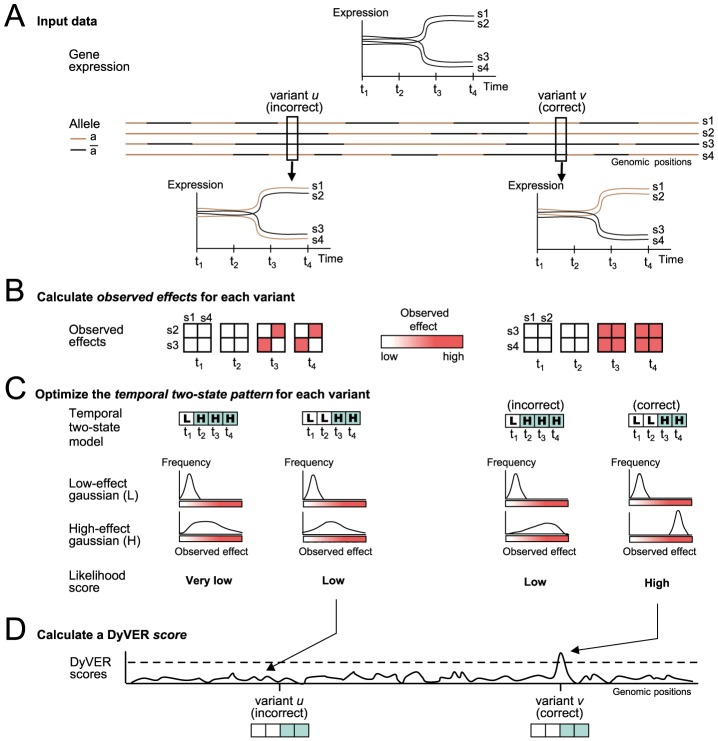
The DyVER algorithm. A methodology for reconstructing genetic associations and their temporal genetic effect patterns from gene expression and genotyping data. (**A**) A cartoon example of input data, including the expression of a single gene over time for strains *s_1_*–*s_4_* (top panel; shown as in **Fig. 1A**), and a typical genotyping of (homozygous) strains carrying either the 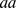 (brown) or 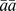 (black) genotype in each genomic position (bottom panel). Correct and incorrect variants (*v*, *u*, respectively) are highlighted. (**B**) Shown are observed effect matrices for each time point from *t_1_* to *t_4_* (red, high-effect size; white, low-effect size). DyVER calculates the observed effects between each pair of strains carrying distinct alleles (strains carrying aa or 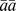 in columns and rows, respectively), using a variant *u* (left) or *v* (right). (**C**) Searching for the temporal two-state model that best fits the data. Shown are four cases, for two possible variants *u*, *v*, and two possible two-state models. The two states are ‘H’ (light blue) and ‘L’ (white) indicating high and low genetic effect, respectively. DyVER's fit of observed effects (high or low) in two Gaussians and the respective likelihood scores are presented in each case. For each variant, DyVER uses an HMM-based dynamic programming to identify its best-likelihood effect pattern. (**D**) A Manhattan plot of DyVER scores. Shown are likelihood ratio scores, called *DyVER scores* (*y-*axis), quantifying each variant (*x*-axis) with its selected temporal two-state model (from **C**). A dashed line indicates the significance threshold, generated using a permutation test.

We devised a new method, DyVER, to identify genetic variants that underlie the expression of genes and their particular dynamic effect patterns. DyVER takes as input the measured transcription response of a gene over several consecutive time points following stimulation and across a cohort, as well as a set of potential genetic variants and their genotyping (**Fig. 2A**). Given a candidate genetic variant with two alternative alleles, DyVER proceeds in three steps (**Methods**): (1) It first calculates the *observed effect* of the variant, namely the difference in gene response between strains carrying the two distinct alleles (**Fig. 2B**). The observed genetic effects are used as data in the subsequent steps. (2) To identify non-linear dynamic shapes of genetic effects, DyVER assumes a ‘digital’ regulatory model that distinguishes two possible states of genetic effects: first, a strong effect of genetic variant on the gene response (denoted the *high-effect state*); and second, a lower (such as zero) effect, or possibly an opposite effect (denoted the *low-effect state*). Several previous methods have employed a two-state model, although not in a dynamic or a genetic effect context [28]. Based on a maximum likelihood approach, DyVER seeks a genetic variant and a sequence of states that best describe the dynamic changes in the size of the genetic effect. For example, if a gene is affected mainly by a variant *v* during a late time interval, DyVER successfully infers the correct effect pattern low→low→high→high for the correct variant *v* as it attains the highest likelihood score (**Fig. 2B and C, right panel**). For incorrect variants, the likelihood scores are typically lower (**Fig. 2B and C, left panel**). DyVER's predicted sequence of states is referred to as the *temporal two-state model*. Finally, (3) DyVER calculates the statistical significance of association for each genetic variant based on a likelihood ratio score that takes as input the inferred temporal two-state model (**Fig. 2D**). We refer to this score as the *DyVER score*. Notably, although DyVER requires synchronous observations in particular time points, it is still possible to apply DyVER on partial observations in each of the time points (**Methods**).

Overall, step 1 allows DyVER to focus on dynamics in genetic effects regardless of the magnitude of transcription response, whereas the discrete modeling in step 2 allows detecting any sequence of spikes up or down in genetic effects. The two-state model from step 2 enhances the performance of the DyVER score (step 3) by allowing a separate parameterization for each of the states. Specifically, to infer an optimal temporal two-state model, DyVER uses a two-state hidden Markov process where the observed effects are treated as the outcome of a sequence of hidden high-effect and low-effect states (step 2; **Fig. 2C**). The corresponding likelihood function consists of two components: (i) the *probability of observed effects* given a certain temporal two-state model; and (ii) the *probability of a temporal two-state model*, which may use a *penalty* factor to prioritize two-state models with a lower number of transitions between states, assuming dependencies among consecutive time points. In the absence of penalty, the order of time points is irrelevant and therefore the predicted two-state model can be viewed as a partition of an unordered group of time points into two sub-groups. The DyVER score exploits this partition for a different parameterization of the (unordered) time points in each of the two states. The addition of the penalty factor makes it possible to avoid an overfitted two-state model that is then given as input to the next step, hence further improving the DyVER score's performance.

### DyVER accurately identifies dynamic genetic effects over time

We compared DyVER's performance to that of five alternative methods. In the first method, the most naïve approach, an ANOVA test is applied at each time point independently and the predicted genetic variant is the one with the most significant (minimal) ANOVA *P* value score. The second method builds on dimension reduction using principal component analysis (PCA): Given *T* time points for each strain as input, it first reduces the *T*-dimensionality of the data into a single dimension by projecting each strain onto the first principal component. Next, it applies an ANOVA test on this one-dimensional data [23]. The third method models dynamics in gene expression as well as dynamics in genetic effect sizes [15]. For comparison, in the fourth method, a linear time term is included as a covariate in the ANOVA test to model dynamic changes in gene expression (without direct modeling of dynamics in genetic effects). Finally, we compared DyVER to a random prediction of association relationships. We called these approaches ‘naïve’, ‘PCA’, ‘detailed dynamics’, ‘expression dynamics’ and ‘random’, respectively. In both DyVER and all compared methods, for each simulated gene, the resulting *P* values were Bonferroni-corrected for the testing of multiple genetic variants. The quality of predicted variants were evaluated using the accuracy metric, defined as the tradeoff between the sensitivity and specificity of revealing genetic variants across different significance cutoffs. The accuracy metric ranges between 0 and 1 for poor and excellent performance, respectively (**Methods**).

To characterize DyVER's ability to reveal dynamic genetic variants and distinguish their effect patterns, we generated synthetic collections of genes that are associated with genetic variants over time. A single synthetic ‘collection’ consisted of 500 genes, 300 of them associated with a genetic variant over time, with two characteristic parameters: (i) the number of time points, and (ii) the effect size (in all cases we used 50 strains and 100 genetic variants). In a complete synthetic ‘dataset’ we generated 72 collections for various numbers of time points and effect size values. Overall, four synthetic datasets were generated in this study, each consisting of a different key class of dynamic effect patterns (see **Methods**): a linear-like pattern (**Fig. 1B**), a single state-transitioning based on a sigmoid function (**Fig. 1C** **, left**), and impulse and multiple-pulse (complex) patterns based on the product of two sigmoid functions (**Fig. 1C**, **middle** and **right**, respectively) [24]. In the following, we first analyze the performance of the DyVER's predicted associations (based on the DyVER score) in the absence of penalty and then present the contribution of the penalty factor.

**Figure 3:**
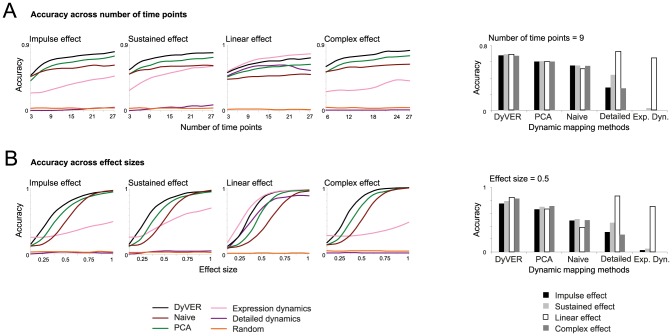
Comparative performance analysis on synthetic data. Shown is the accuracy measure (scatter plots, left) and an example (histograms, right) across compared methods and different synthetic data parameters. Left: The accuracy measure (*y*-axis) using different patterns of genetic effects (impulse, single state-transitioning (sustained), linear, and complex sub-panels). Results are shown over genes that were measured in different numbers of time points (measures were averaged over effect sizes; *x*-axis, **A**), or over genes of different effect sizes (averaged over time points; *x*-axis **B**). Plots depict six alternative mapping methods (color coded). Right: Examples of performance (*y*-axis) using the four different dynamic effect patterns (color coded) across various methods (*x-*axis) for nine time points (**A**) or for genetic effect size 0.5 (**B**). The plots indicate that for non-linear genetic effect patterns, DyVER has an advantage over existing methods.

DyVER showed good accuracy in all non-linear dynamic effect patterns (0.5 penalty; **Fig. 3**). **Fig. 3A** presents the accuracy metric for synthetic datasets of varying numbers of time points. Accuracy values are averaged across the eight collections of distinct effect size. In all non-linear dynamic effect patterns, DyVER displayed the best accuracy in all tested time points ranging between 3 and 27, with improved accuracy for a larger number of time points. Importantly, although DyVER was not designed for linear-like effect patterns, it still attains the second-best performance for this case. The ‘expression dynamics’ approach yielded the most accurate predictions for the linear case, but attained poor results in the non-linear case. The tradeoff between sensitivity and specificity in the accuracy measure across the different methods is further demonstrated in **Figure S1A** and **B**. Results were similar for varying effect sizes (**Fig. 3B** **and Figure S1C** and **S1D**) and for an additional synthetic dataset that is based on prototypical effects in *C. elegans* (**Methods**; **Figure S2**). Furthermore, although DyVER's accuracy is reduced in the case of missing data, it is still notably high in comparison to alternative methods (**Figure S3**). Taken together, our results indicated that DyVER performs well on a broad range of genetic effect patterns.

We next aimed to characterize DyVER's applicability to short-term steady state of high genetic effects. To tackle this goal we compared two synthetic impulse datasets, both consisting of 27 time points across various effect sizes. For all genes, the short-impulse dataset consisted of a high-effect steady state of short duration (five time points), whereas the long-impulse dataset consisted of a high-effect steady state of long duration (fifteen time points). **Figure S4A** records the performance of DyVER compared to the five alternative methods on the short-impulse and the long-impulse datasets, and clearly shows that DyVER outperformed the alternative procedures when genetic influences were acting in short impulses, even with low-effect sizes.

The performance of both DyVER and the alternative methods declined when applied on a short impulse compared to a long impulse of genetic effects, but notably, the performance reduction was lowest with DyVER (**Figure S4B**). For example, for high-effect sizes (0.625), the sensitivity of DyVER is 0.7 and 1 with short and long impulses, respectively. The sensitivity of PCA, in contrast, is respectively 0.47 and 1 with short and long impulses for the same effect size. Thus, even when genetic variants acted during short time intervals, DyVER still performed relatively well. This was unlike the alternative methods, whose performances were drastically reduced even for relatively high-effect sizes.

DyVER predicts a temporal two-state model, which may provide insights concerning the timing of changes in genetic effects (**Fig. 2C**). To evaluate the quality of this prediction, we compared the ‘ground truth’ (simulated) models against the inferred two-state models. We chose to work with the established error rate statistics, defined as the number of erroneous two-state models expressed as a fraction of the total number of significant correctly predicted variants. We called this metric a *two-state pattern error rate* (in short, error rate), and calculated it both for the case of stringent (exact) matching or flexible (non-exact) matching between the true and inferred models (**Methods**). In both cases, we found that DyVER performs well in predicting two-state models, where the flexible case outperforms the stringent case, as expected. For example, using single state-transitioning patterns with nine time points, effect size 0.75, significance cutoff 0.001 and the absence of penalty (probability of transition 0.5), the stringent and flexible error rates are 0.41 and 0.33, respectively (**Figure S5**). The error rate increased with decreasing penalty (e.g., for transition probabilities of 0.01 (high penalty) and 0.5 (no penalty), stringent error rates are 0.32 and 0.41, respectively). As expected, error rates rose when a higher statistical significance cutoff (0.05) was used, whereas the gap between the error rates for different significance cutoffs remained relatively constant when the penalty increased. Results obtained for other effect sizes were similar.

Collectively, our results indicated that DyVER outperforms extant methods even in the absence of penalty and the presence of missing data (**Fig. 3**, **Figures S1–S4**), and that these performance can be even enhanced by the addition of a penalty component (**Figure S5**). These results hold when the complexity of dynamic effect patterns is relatively low, as in the case of genetic effects in biological data (*e.g*., **Figure S6**).

### A catalogue of non-linear genetic effect patterns in yeast response to rapamycin

**Figure 4:**
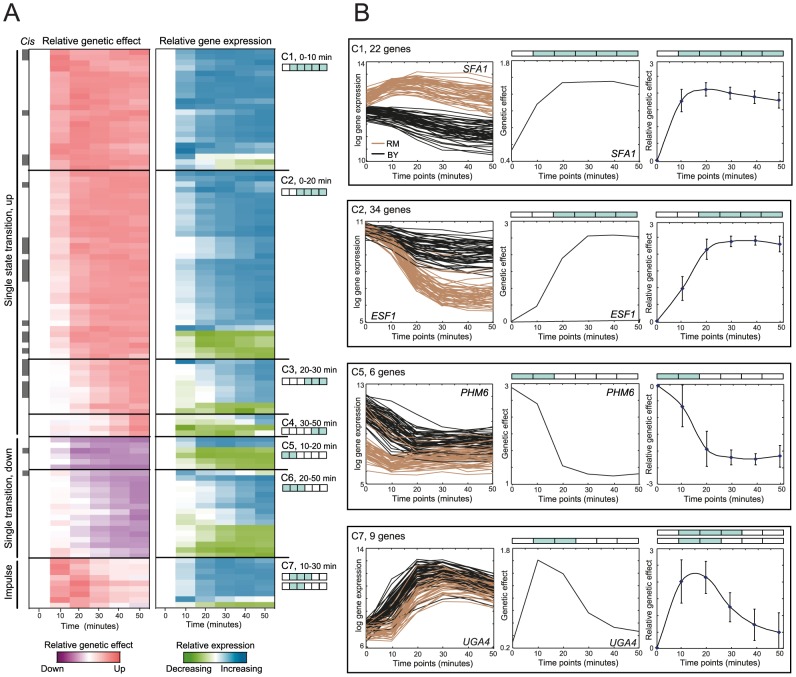
A catalogue of dynamic, non-linear genetic effects in gene response following rapamycin treatment in yeast. (**A**) Genetic effect profiles (left) and gene expression profiles (right) at six time points following rapamycin treatment (columns) for all genes identified by DyVER. Genetic effect values are the average increase (red) or decrease (purple) in effect size relative to non-stimulated cells (log-scaled). Gene expression values are the average increase (blue) or decrease (green) in gene expression relative to non-stimulated cells (log-scaled). *Cis*-associated genes are marked in gray (left color bar). Genes are partitioned into seven groups (C1–C7) based on their temporal two-state model (two state cartoons, shown as **Fig. 2c**, right; four singleton genes are omitted). (**B**) Four temporal two-state model groups C1, C2, C5, C7 (top to bottom). Left and middle panels: representative genes in each group. Left: gene expression of a representative gene (*y*-axis, log-scaled) across time points (*x*-axis). Each curve represents a different segregant, color coded by the best genetic variant found using DyVER (BY/black, RM/brown). Middle: genetic effect profiles of the representative gene, averaged across strains (log-scaled, *y*-axis) at each time point (*x*-axis). Right: shown are mean genetic effects (relative to non-stimulated cells, log-scaled; *y*-axis) and standard deviation (error bars) across time points (*x*-axis) for a certain group of genes.

We applied DyVER in an unbiased manner (without penalty) to the available dataset of 95 yeast segregants that were stimulated by rapamycin and profiled at six time points (**Methods**) [27]. DyVER predicted 351 associations to 145 distinct variants (false discovery rate [FDR] 6%). Of these 351 associations, 145 had highly significant dynamic associations (15% FDR, **Table S1**, **Methods**) and 105 of them showed non-linear genetic effect patterns (**Fig. 4**). In agreement with previous findings [2], [11], our results suggest that non-linear associations are prevalent: of the eight previously known causal genes, six were found to have an association with at least one target gene exhibiting a non-linear genetic effect pattern (**Table S2**). Correlations among genetic effects of consecutive time points were much larger than correlations between non-consecutive time points [*P* value <10^−15^ (Wilcoxon test)], justifying our ‘memoryless’ Markov assumption that the next time point is mainly dependent on the current time point (**Figure S7**). The 105 genes carrying non-linear effect patterns were partitioned into groups based on their predicted two-state pattern (**Table S1**); seven two-state pattern groups (C1–C7) were created, each including at least two genes (**Fig. 4A and B** **)**.

The partition revealed three prototypical non-linear genetic effect patterns (**Fig. 4A**), including (i) a single upward spike followed by a sustained high level of genetic effect (70 genes in C1–C4). These different groups were characterized by distinct timing of a state-transitioning, including an abrupt change in early time points (0–10 min, C1), as well as an intermediate-early (0–20 min, C2) and intermediate-late (20–30 min, C3) single state-transitioning. For example, *SFA1* and *ESF1* (in groups C1, C2) demonstrate a sustained genetic effect with a state transition at 0–10 and 0–20 minutes after rapamycin stimulation, respectively (**Fig. 4B**). In the case of the four genes exhibiting a late state-transitioning (at 30–50 min, C4), a sustained new level of genetic effects might occur at later time points that were not measured in the current dataset [27]. (ii) A single downward spike of genetic effect (C5–C6, 22 genes). In group C5, we observe an abrupt downward spike in 10–20 minutes followed by a sustained low level of genetic effect (for example, *PHM6*, **Fig. 4B**). Group C6 represents a delayed gradual single state-transitioning during 20–50 minutes. (iii) An impulse of high genetic effect at 10–30 minutes after treatment (9 genes in C7, e.g., *UGA4*, **Fig. 4B**). Overall, the single state-transitioning patterns were over-represented, whereas complex patterns of genetic effects were rare (1 gene, *YER053C-A*) and were under-represented [*cis*: *P* value <10^−19^, *trans*: *P* value <10^−50^ (*t*-test), (**Figure S6A**)]. Our findings of rare complex patterns in yeast parallel similar observations in the mouse (**Methods**, **Figure S6B**); Yet, the particular shape of effect patterns may differ between biological systems (**Figure S8**).

### High-order coordination suggests that dynamic effect patterns are an emergent property of genetic variants

**Figure 5:**
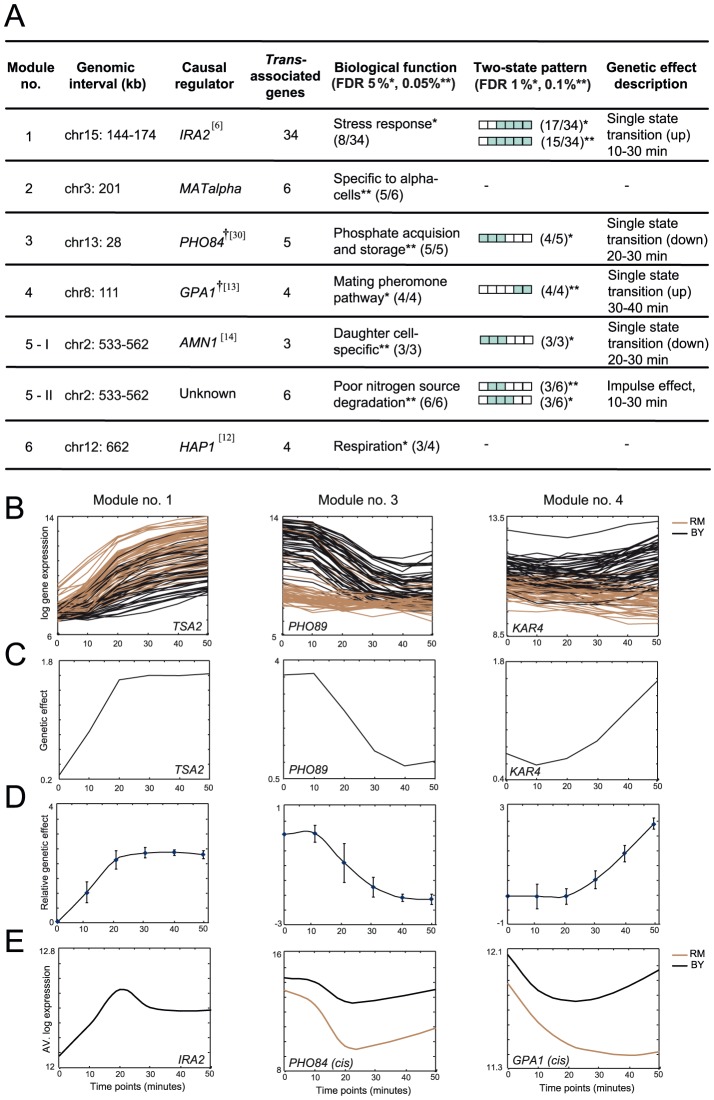
Co-associated genes typically share a similar pattern of genetic effects over time. (**A**) Six gene modules (column 1), constructed on the basis of a shared *trans-*associated genetic variant (a genomic interval; column 2), are listed together with their known causal gene, if available (column 3; ^†^ *-cis*-associated causal gene, references are in parentheses) and the number of associated genes in a module (column 4). Significant enrichments in biological processes are detailed in column 5. Significant enrichments of temporal two-state patterns in each module are presented together with the description of these enriched patterns (columns 6 and 7, respectively). (**B−E**) Gene expression and genetic effects in modules nos. 1 (left), 3 (middle) and 4 (right). Gene expression (**B**) and genetic effect (**C**) of representative genes, as well as genetic effects of an entire module (**D**); plots are shown as in **Fig. 4B**. (**E**) Average gene expression (*y*-axis) at six time points (*x*-axis) for the known causal gene of each module. For *cis*-associated causal genes (modules nos. 3 and 4), brown and black indicate strains carrying the RM and BY alleles, respectively. The plots demonstrate the good match between the timing of abrupt changes in causal genes (**E**) and the timing of alterations in the observed genetic effects of their associated target genes (**D**).

We next explored the pleiotropic *trans*-acting variants that arise from this analysis. Using DyVER's predictions we organized the genes into six co-association modules, each containing a group of (at least two) genes with the same *trans*-associated variant (**Fig. 5A and B** **)**. Functional enrichment strongly related all six modules with specific biochemical pathways. For example, the entire module no. 3 consists of genes that play a role in uptake of phosphate (P_i_) from extracellular sources and its accumulation in vacuoles (5 of 5 genes; **Fig. 5A and B** **, Figure S9A**). The module's validated causal gene is *PHO84*, a high-affinity phosphate transporter that carries a missense mutation in one of the parental strains (**Figure S9B**) [29], [30]. The nine genes in module no. 5 carry two distinct functionalities and are therefore treated as two distinct sub-modules, no. 5-I and no. 5-II (three daughter cell-specific genes and six poor nitrogen source degradation genes, respectively, **Fig. 5A**).

Next we examined whether module genes show characteristic temporal effect patterns. On analyzing the modules we found that modules nos. 1, 3, 4, 5-I and 5-II relate to a specific prototypic temporal genetic effect pattern, whereas the remaining two modules (nos. 2 and 6) are more general and show several distinct patterns (**Fig. 5A**). For example, module no. 1 contains 34 genes, 32 of which have an upward spike (a single state transition) of genetic effect at 10–30 minutes after rapamycin stimulation [FDR 0.01 (hyper-geometric test)]. As another example, module no. 3 contains five genes, all showing a downward spike of genetic effects at 10–30 minutes after stimulation. Specifically the downward spike occurs either 20–30 minutes after stimulation [4 genes, FDR 0.01 (hyper-geometric test)] or 10–20 minutes after stimulation (1 gene, **Fig. 5A–C** **, Figure S9C**). Overall, we found four modules with over-represented patterns of single state-transitioning at specific time points (nos. 1, 3, 4 and 5-I) and one sub-module of an impulse effect pattern (no. 5-II). The observed coordination of temporal genetic effects does not necessarily reflect a coordination of transcription responses (**Figure S10**). In previous reports, baseline expression levels were used to identify eight genetic variants underlying similar modules (**Table S2**), but the coordinated temporal genetic effects and the timing of upward or downward spikes of genetic effects were not characterized.

A plausible explanation for the ‘shared variant, shared temporal genetic effect pattern’ hypothesis is that the same molecular mechanism underlies both inter-individual variation and the dynamics of genetic effects. In such cases, the dynamic pattern of effect is an attribute of the underlying regulatory mechanism (rather than of the target genes), probably owing to temporal changes in the influence or activity of the regulatory mechanism. This hypothesis is further supported by the consistency in the timing of state transitions in module genes and their underlying (known) causal genes (**Figs. 5D** versus **5E**): The *trans*-associated causal gene of module no. 1 (*IRA2*) attains a sustained-like pattern of gene expression that resembles the temporal genetic effect pattern of its target genes (**Fig. 5D and E**, **left**). The *cis*-associated causal genes in modules nos. 3 and 4 (*PHO84* and *GPA1*) exhibit drastic changes in their transcription response at the same time point at which there is a (downward or upward) spike in the genetic effect of their target genes (20–30 and 30–40 min; **Fig. 5D and E**, **middle** and **right**, respectively).

### A novel pleiotropic variant acting on the timing of initiation of transcriptional response

**Figure 6:**
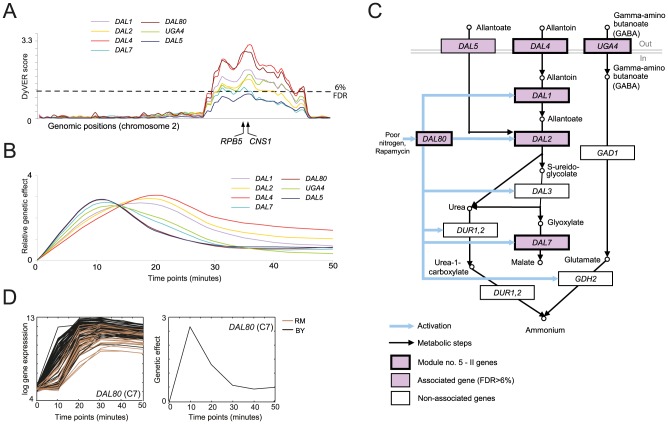
A genetic variant acting on the timing of response of the poor nitrogen-source degradation pathway (module no. 5-II). (**A**) The genomic interval underlying module no. 5-II residing in Chr2: 533–562 kb. Shown are DyVER scores (*y-*axis) across the genomic positions in chromosome 2 (*x*-axis) for seven associated genes (color coded; the module includes only those six genes that cross the FDR 6% threshold). Positions of two potential causal variants, *RPB5* and *CNS1*, are marked below. (**B**) Genetic effects, relative to non-stimulated genetic effects (*y*-axis, log-scaled) for different associated genes from **A** (color coded) at six time points (*x*-axis). The plot depicts the short impulse of high genetic effect in all associated genes. (**C**) Module genes, in the context of the poor nitrogen-source degradation pathway. Enzymes are shown as color-coded rectangles (bold-pink/module genes, pink/associated genes, white/non-associated genes). The pathways show the uptake of poor nitrogen sources (allantoate, allantoin, and GABA) and their degradation into ammonium. (**D**) A representative gene. Expression profiles (left) and genetic effects (right, *y*-axis) of *DAL80*, a gene in module no 5-II, during response to rapamycin (*x*-axis). Shown as in **Fig. 4B** but using a marker near the *RPB5* gene (marked in **A**).

The poor nitrogen source degradation system (module no. 5-II) demonstrates the ability of our method to reveal novel associations acting on the timing of response and affecting an entire cellular pathway (**Figs. 5** **,** **6**). During growth on relatively poor nitrogen sources (allantoate, allantoin, and GABA), yeast cells activate premeases responsible for uptake of nitrogen sources and further increase the expression of enzymes that participate in degradation of poor nitrogen sources for the generation of ammonia. Exposure to the TOR inhibitor rapamycin also leads to the same nitrogen-regulated response [31]. Module no. 5-II consists of six of the twelve genes in the allantoin, allantoate and GABA degradation pathways, with all six genes having a significant impulse effect pattern (*DAL1, 2, 4, 7, 80* and *UGA4*; **Fig. 6A–C**). An additional gene in these pathways, *DAL5*, is weakly associated using the same impulse pattern at the same genomic position (**Fig. 6A–C**).

The impulse pattern reflects a difference in the timing of initiation of response among the strains carrying the RM and BY alleles in Chr2: 533–562 kb. For example, strains carrying the BY allele showed early up-regulation of *DAL80* in response to rapamycin, which was already detected at 10 minutes after stimulation. The RM-carrying strains, in contrast, showed a clear delay in response to rapamycin, but all strains reached a similar expression level by 30 minutes after stimulation (**Fig. 6D**). The underlying genetic variant acting on the timing rather than on the magnitude of response has not been previously documented. In the genomic interval (Chr2: 533–562 kb), two genes (*RPB5, CNS1*) have temporal transcription profiles that match the expected early impulse of high genetic effect, the promoter of five genes (*RPB5, CNS1, ADH5, RTC2, YBR144C*) is bound by nitrogen-related transcription factors [32], and four genes (*RPB5, CNS1, ADH5, RTC2*) were previously reported in nitrogen-related cellular processes (**Figure S11**). These criteria therefore suggest that *RPB5* or *CNS1* are two leading candidates in module 5-II.

## Discussion

In this work we present the DyVER computational algorithm for identifying genetic variants that lead to dynamic changes in genetic effects. DyVER was tailored to identify abrupt changes in the levels of genetic effects, which may provide valuable information about the timing of alterations in the particular regulatory mechanisms interacting with the underlying genetic variant. In comparison with other approaches, DyVER attained the most accurate identification of non-linear genetic effect patterns, even in the absence of penalty (**Fig. 3** **, Figures S1–S4**), likely due to (i) a focus on genetic effects rather than on modeling the original phenotype values, and (ii) the prior knowledge about the separation of the time points into two distinct groups that differ in their observed effects (encoded in the temporal two-state model), thus allowing a different parameterization for each of these groups.

DyVER is using an HMM-based model for revealing genetic variants acting on time-series gene expression data. HMM modeling has been applied in various contexts, but not for the case of direct identification of underlying genetic variants. For example, HMM has been utilized for the identification of CNVs or haplotypes [33], [34]. Alternatively, an existing method was mainly focused on revealing differential expression between conditions using an HMM approach [26]. DyVER extends this method by providing a statistical genetics *P* value score and by allowing a number of parameters that is not increasing with the number of strains.

Our method opens multiple directions for future investigations. First, it is important to extend DyVER for the case of outbred heterozygous population, including human. In the current study, DyVER was designed for the case of a inbred (homozygous) strains that are common in genetic studies (e.g., in yeast, nematode, fly, mouse and rat) due to several major advantages: first, inbred strain enable controlled stimulations, and second, they avoid major challenges that are common in human genetics, including haplotype analysis, rare variants and uncontrolled variables. Future extensions may generalize the method for the heterozygous case, possibly by calculating genetic effects between each pair of genotypes (rather than between the only two possible genotypes as in the homozygous case), requiring to add additional one or two Gaussians within each of the model states. Second, the usage of a our probabilistic model leads to several limitations: the number of states should be specified in advance; we only capture correlations between sequential time points but cannot capture higher-order correlations among time points; and we generally assume that the probability of a time point is independent of the probabilities of its neighboring time points. Future improvements that handle more than two states and a more sophisticated probabilistic graphical model [35] may therefore enhance DyVER's performance. Third, DyVER relies on at least a few synchronized strains in each of the time points. Although DyVER allows missing data and possibly different strains in different time points (**Figure S3**), it still cannot be applied on non-synchronous data (as in [11]). Data imputation methods can potentially enhance the DyVER analysis beyond this synchronization requirement.

Building on the DyVER approach, we analyzed temporal gene expression patterns following rapamycin treatment in yeast segregants. Our analysis identified 105 genes exhibiting significant non-linear genetic effects over time, 56 of them are well-established associations (in modules 1,2,3,4,5-I and 6), and the remaining genes are new candidates for future experimental investigations (e.g., **Fig. 4B**). For example, our study suggests a novel genetic variant residing in chr2: 533–562 kb as the underlying regulator of the timing of upward spikes in gene expression after rapamycin treatment. Reassuringly, this regulator acts primarily on genes that play a role in poor nitrogen source degradation (6 of 6 genes, module 5-II, **Fig. 6**).

The application of DyVER in yeast provided several novel insights that were mainly attained due to the unique capability of DyVER to classify associations based on their optimized temporal effect patterns. First, we use the temporal effect pattern to automatically organize the genes into clusters based on their predicted patterns (**Fig. 4A** **and Figure S12**). This organization is substantially different from previous studies [2], [11] that have grouped time-series associations only manually. Based on this clustering, we found that abrupt single state-transitioning and impulse patterns occur in certain prototypical time points. In particular, DyVER identified an upward spike of genetic effect at 0–10, 0–20, 20–30 and 30–50 minutes (22, 34, 10 and 4 genes, groups C1, C2, C3 and C4, respectively); a downward spike followed by a new sustained low level of genetic effect (6 and 16 genes at 10–20 and 20–50 minutes, groups C5 and C6, respectively), and a single pulse of high genetic effects (9 genes, group C7, **Fig. 4**).

Second, many studies have shown that groups of co-associated genes also share similar functionalities. Interestingly, our results indicate that such co-associated genes typically share not only a similar functionality, but also a similar predicted pattern of temporal genetic effect (**Fig. 5**). One plausible explanation is that a causal regulator typically alters its functionality during its response to stimulation; therefore, a genetic variant interacting with such a regulator is likely to affect its target only during those time intervals in which the regulator is functional. Based on this rationale, the temporal effect patterns in target genes may uncover the temporal dynamics of their causal regulatory mechanisms. Thus, DyVER's characterization of temporal effect patterns, which are probably a property of the causal regulatory mechanisms, may provide a starting point for improved identification of causal genes. For example, it might be possible to pinpoint a causal gene in a genomic interval based on its predicted dynamics over time (as demonstrated in **Fig. 5D and E** **and Figure S11C**). Furthermore, it may be possible to discriminate between two genetic variants differing in their dynamic over time, even when these variants are co-localized at a nearby genomic position (as in module nos. 5-I and 5-II, **Fig. 5A**).

Taken together, our results highlight the utility of studying temporal genetic effect patterns to discover and characterize dynamic causal regulators. The next step is to extend and apply our approach to map genetic effects in transcriptome of a wide range of mammalian cell types.

## Material and Methods

### The DyVER algorithm

#### Input data

For simplicity of presentation, we assume synchronous data across all time points and strains. We will show later, however, that this requirement can be relaxed. DyVER takes as input the expression of a gene across *n* strains and *T* time points. The gene's profile at time point *t* is denoted 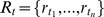, where 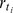 is the (log-transformed) expression level in the *i*-th strain and *t*-th time point (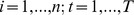). The input also consists of *K* genetic variants 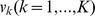 that are genotyped across the population. For homozygous recombinant inbred strains, the genotyping of each strain in each genetic variant 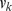 is either 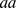 or 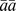. For convenience of description, a variant 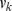 partitions the strains into two groups, 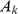 and 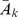, consisting of 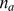 and 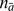 strains carrying its 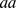 and 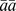 genotypes, respectively.

#### Calculating the observed effects

Typically, the expression of a gene is affected mainly by stimulation and is further modulated by a genetic variant. To successfully detect the minute effects of genetic variants, it is necessary to remove the confounding effects of the stimulation. Hence, our first step is to calculate the differences between expression values of strains carrying different alleles. We term these differences the ‘*observed effects*’, and we use this collection of observed effects in the rest of the method. For example, when a genetic variant acts, its observed effect values are usually high (e.g., time points *t_3_* and *t_4_* in variant *v*, **Fig. 2A and B**); much lower effect values are observed in the absence of influence (e.g., time points *t_1_* and *t_2_* in variant *v*, **Fig. 2A and B**) or when a wrong genetic variant is tested (e.g., variant *u*, **Fig. 2B**).

More precisely, given a time point *t*, the putative effect of a variant 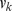 on two strains *i,j* carrying its 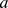 and 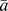 alleles is: 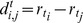



Where 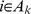 and 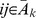. Overall, the collection 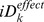 consists of 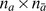 observed effects of variant 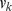 at any time point *t*:
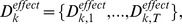


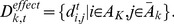
(1)


Since DyVER assumes that the variance of the genetic effects is not changing over time, variance stabilization methods should be applied before or after calculating the observed effects. In this study we first normalized the log-transformed expression level in each time point so that the variance in each time point and each allele is fixed (but the mean values remain unchanged). The observed effects were calculated only after this transformation.

#### Formalizing the likelihood of the data

Our digital model assumes that at each time point a genetic variant may attain one of two states: either the *high-effect state* (‘*H*’), reflecting the observed presence of a high genetic effect, or the *low-effect state* (‘*L*’), reflecting either small effect, an opposite effect, or the absence of effect. We denote by 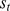 the state of a variant at the *t*-th time point 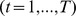. A particular *sequence of states* (also referred to as a candidate *temporal two-state model*) is 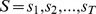 where state 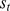 takes one of two values, *H* or *L*. We model the dependencies among time points as a first-order Markov chain, assuming 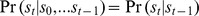. We use a fixed probability of transition, called *penalty*: 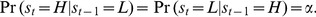
(2)


The lower the 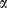, the higher the penalty, whereas no penalty is applied when 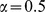.

The overall *probability of a temporal two-state model* 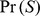 is calculated across the time points, starting at the first time point with the *initial state probability* 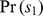 as follows:
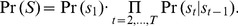
(3)


Assuming 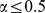, this formalization reflects the desired dependencies among time points: the higher the number of state transitions, the lower the probability of a temporal two-state model.

Assuming that the temporal two-state model of a variant 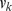 is 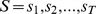 (where 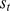 is in either the *H* or the *L* state), the probability of measuring such observed effect values is
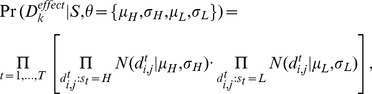
(4)referred to as the *probability of observed effects*. In both states *H* and *L* the effect is modeled with mean Gaussian noise of 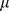 with a standard deviation 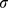. Each of the states, however, may attain distinct parameter values: 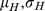 are used for the *H* state and 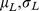 for the *L* state. Notably, the parameters for all time points in the same state are shared, opening the way to a large number of time points without increasing the number of model parameters.

Collectively, given a temporal two-state model *S*, model parameters 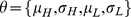 and a variant 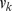, the *likelihood function* for a candidate gene is:
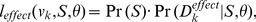
(5)where 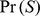 and 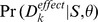 are calculated as in equations 3 and 4, respectively.

#### Learning the temporal two-state model using a likelihood function

For each candidate variant 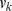, DyVER searches for the temporal two-state model 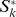 and model parameters 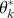 that maximize the likelihood function: 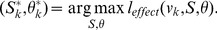
(6)


Notably, the model can be viewed as a two-state Hidden Markov Model (HMM) [36] consisting of a sequence of high-effect and low-effect states that are hidden, where the observed effects at each time point are sampled from a Gaussian probability distribution. We can therefore use existing HMM methods for the maximization process. In particular, we use the Baum-Welch algorithm [37], which utilizes expectation maximization steps applied on an HMM model to iteratively improve the hidden sequence of states (temporal two-state model) and the parameters of the model from the hidden states. In all cases, our initialization is a pattern of low-effect states using distinct average values in each gaussian; we evaluated that in 93% of the cases, a single optimization with such a single initialization equals the maximal likelihood that could be attained by testing 1000 random initializations. After the optimization process and without loss of generality, the state whose absolute 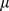 value is higher (lower) is referred to as the H (L) state. Notably, since both 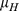 and 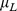 are not restricted to any particular value, the model may capture various dynamic changes in effect size, including inversions.

#### Statistical evaluation

Naïvely, the maximal likelihood score 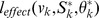 can be used to search across all candidate genetic variants that may associate with a gene. However, such a score cannot help us make a judgment about the statistical significance of such a hypothesis. Here we want to know whether the observed effects of a candidate variant 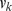, assuming its temporal two-state model 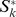, can be attributed to chance. In particular, to share parameters of observed effects across all time points in each of the states (according to 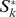), the test is applied directly on observed effects (rather than on the original expression levels whose parameters cannot be shared based on 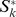). We distinguish two populations of differences between expression values of distinct strains: first, the population of observed effects 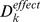, consisting of differences among strains carrying distinct alleles (as defined in equation 1); and second, a background population 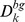, consisting of differences among strains carrying the same allele:
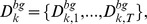


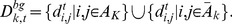
(7)


The null hypothesis 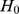
assumes that a variant 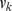 has no effect; thus, a single model can represent both samples 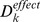 and 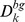. The alternative hypothesis 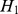 is that the variant has a dynamic effect on gene response, and thus that 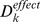 and 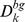 should not be modeled together. The test statistic is the ratio:
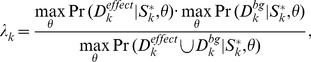
(8)where 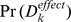, 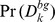, and 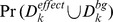 are calculated as in equation 4 but for different datasets. We call the test statistic 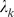 the *DyVER score*. The significance level of the score is evaluated by repeatedly permuting the labels of strains. We calculate an empirical *P* value, defined as the proportion of permutation tests for which the DyVER score is larger than the observed (non-permutated) score. **Figure S13** indicates that the DyVER score *P* value is indeed well-calibrated using a Q-Q plot analysis. Reported *predicted association(s)* are those with significant DyVER scores.

Notably, in standard genetic methods, the null model's input dataset is similar for different variants. For example, the null model of a regression analysis utilizes the same data values but with variant-specific predictors; the null model of an ANOVA test is variant-independent. In the same sense, the null model of the DyVER score is based on 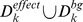 that is calculated based on all possible pairs of strains regardless the variant under study. Alternative scores – focusing solely on 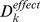 and omitting the 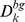 component (e.g., **Figure S14**) – have not been used throughout this study since they are prone to attaining 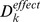 datasets with an entirely different set of strains pairs for different variants.

In a post-processing step, it is also possible to evaluate the dynamic nature of DyVER's predicted associations. To this end, the *dynamic association score* may test the significance of the difference between the observed effects of the high-effect and the low-effect states (a *t*-test *P* value score). The partition into high-effect and low-effect states is determined by the predicted temporal two-state model (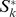 from eq. 6). *Predicted dynamic associations* are those predicted associations for which there is a significant dynamic association score. Whereas the DyVER score is a statistical genetics score that identifies an underlying genetic variant, the dynamic association score evaluates temporal changes for this variant.

Since the calculation of observed effect and likelihood measures are calculated in each time point independently of its neighbors (eqs. 1–6), and since our scoring scheme relies only on these measures, thus the input data in each time point may consists of a different population of strains with a different population size. In particular, as long as the input data consists of multiple synchronous strains in each time point, DyVER allows missing data without a requirement for data imputation. DyVER's executable and source code, including an option for an additional imputation based on flanking time points of the same strain, can be downloaded from csgi.tau.ac.il/dyver.

### Synthetic data

To generate synthetic data we first generated 50 strains carrying 100 genetic variants, sampling one of the two alleles with equal probabilities. A single synthetic collection consists of 500 genes, of which 300 are associated with a certain variant over T time points. Overall, for a single dataset we generated 72 collections, constructed for all combinations of eight possible ‘effect sizes’ (defined below, ranging between 0.125 and 1) and nine different numbers of time points (ranging between 3 and 27). In all cases, the low-effect state represents the absence of effect (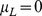) and the high-effect state represents the presence of an effect (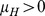), where 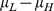 is the effect size. 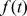 is the simulated observed effect, which is generated by sampling 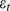 from a Gaussian distribution 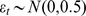. A dataset was constructed for each class of temporal effect patterns. For a single state transition effect pattern (here, sustained) we used a sigmoid function:
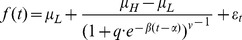



Where 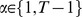, q = v = 0.5 and 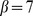. For an impulse effect pattern we used the product of two sigmoid function with five parameters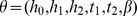 [24], where 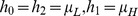, 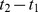 is the length of the impulse effect (here, 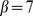):
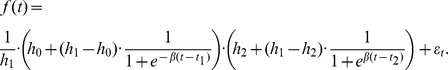



To generate the complex pattern for T time points we concatenated two impulse patterns, each for T/2 time points. For the dataset of linear effect patterns, observed effects are sampled from a linear function: 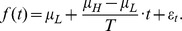



For the purpose of comparing predicted to gold-standard temporal two-state models (**Figure S5**) we generated a different collection of synthetic sustained dataset as follows: we first generated the temporal two-state model by sampling from the corresponding distribution (from equation 3) with 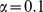. The observed effects were then generated by sampling from the corresponding Gaussian distribution 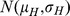, 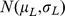, where 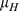 and 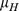 are the mean of the high- and low-effect state, and 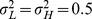. To generate an input with a percentage of *k%* missing data, in each time point, we omitted the information for *k%* randomly selected strains (thus, each time point consists of a different list of strains).

An additional synthetic dataset was created similarly to the above datasets, but using previously published functions in *C. elegans* [11]. For each of the 300 associated genes in this synthetic dataset, we first randomly chose a function out of the 18 functions that were published in *C. elegans*; the observed effects were then sampled from this selected function.

The compared methods were implemented as follows. In the ‘naïve’ method we assumed a simple fixed effect model on each time point independently, 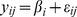, where 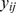 is the observed expression level for strain *j* carrying genotype *i*; 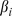 is fixed effect of genotype *i* and 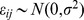. The most significant (minimal) ANOVA *P* value score is taken as the resulting *P* value. In the ‘PCA’ method, we project the *T*-dimensionality of each strain into the first principal component and then applies an ANOVA test assuming a fixed effect model 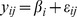 where 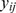 is the first principal component for strain j carrying genotype *i*; 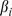 is the fixed effect of genotype *i* and 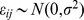 (the first principle component was chosen since it performs better than the consecutive components, see **Figure S15**). For the ‘expression dynamics’ method, we used the model 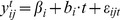 where 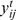 is the observed expression level in time point *t* for strain *j* carrying genotype *i*, 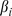 and 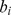 are two fixed effects for genotype *i* and 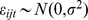. The formulation was implemented using the *lme4* R package. In all cases above, an *F*-test was used to test the model. For the more sophisticated ‘detailed dynamics’ method, we use the *longGWAS* R package that is part of its original publication [15].

### Performance analysis using synthetic data

For each synthetic dataset, DyVER was applied to predict a genetic variant using the DyVER score (*P* values were Bonferroni-corrected for multiple variants). To quantify the ability to correctly predict such genetic variants, we define the accuracy measure. Genes are split into two groups: one contains genes that are associated with a genetic variant, and the other contains the remaining, non-associated genes. A mapping method may provide a negative prediction (i.e., a non-significant *P* value for all candidate variants), or alternatively, a positive prediction of either the correct variant or an incorrect variant. We define true positives as associated genes whose correct genetic variant is predicted with a significant *P* value. True negatives are non-associated genes that were not significantly associated with any variant. False negatives are associated genes that were not significantly associated with any variant. Finally, false positives are defined as erroneous significant predictions as a result of two possible scenarios, either a non-associated gene that is wrongly predicted to be associated with a certain variant, or alternatively, an associated gene whose predicted variant is incorrect. We adopt the standard formulations for sensitivity (number of true positives out of the total number of positives) and specificity (number of true negatives out of the total number of negatives).

Similarly to a standard ‘Receiver Operating Characteristic’ (ROC) analysis, we can plot the sensitivity against the 1-speificity across different *P* values, providing an overall view of the performance of the method: the higher the curve, the better the *accuracy* (defined as the area under the curve). Notably, using a standard sensitivity definition, sensitivity should increase with higher *P* value thresholds. In contrast, using our definition of sensitivity, it is dependent on the particular predicted variant. Thus, even with a very high *P* value threshold and many affected genes, the sensitivity of a random algorithm might remain close to zero. The accuracy therefore ranges between 0 (for a random prediction) and 1 (for a perfect prediction).

Finally, to quantify the ability of DyVER to correctly predict the temporal two-state model, we define the *two-state pattern error rate* (shortened to *error rate*) as the number of wrongly predicted temporal two-state models expressed as a proportion of the total number of (significant) correctly identified variants. We test two different rules for matching between the simulated and predicted model. In the stringent case, we require a fully correct two-state model, and in the flexible case, we require correct transitions between states but allow incorrect timing of transition.

### Yeast and mouse dataset

We applied DyVER to genotyping data and gene expression data that were monitored during six time points following exposure to rapamycin in 95 yeast segregants and their two parental yeast strains: BY4716 (BY) and RM11-1a (RM) [27]. DyVER was applied to the log expression of 2700 genes with the highest difference between the BY and RM parental strains. To ensure that the biological results are unbiased, DyVER was applied with penalty 0.5. Multiple testing was controlled as follows: DyVER score *P* values were first Bonferroni-corrected for multiple variants; the corrected DyVER score *P* values were then controlled for multiple testing of genes (FDR 6%). We then further filtered the genes based on the dynamic association score (FDR 15%). In total out of 2700 genes, we obtained 351 (13%) predicted associations (based on the corrected DyVER score *P* value) and 145 (5.3%) predicted dynamic associations (based on the dynamic association score). Next, we further removed 40 genes carrying linear-like patterns, based on strong correlation with a linear model (r>0.95) and more than 5% change in genetic effect in any two consecutive time points (**Table S1**). The partition into groups was generated automatically according to DyVER's predicated two-state model (**Figure S12**).

In addition, we applied DyVER to genotyping data and log gene expression data of 403 genes that were monitored using a meso-scaled technology during three time points following exposure to lipopolysaccaride in 45 mouse BXD strains [2]. Of the 403 genes, 14 genes (3.4%) were identified as significant dynamic associations (FDR 10%; **Figure S6B**).

## Supporting Information

Figure S1 **Comparative performance analysis on synthetic data.** Scatter plots for various performance measures (*y*-axis) of six alternative mapping methods (color coded) over genes that were measured in different numbers of time points (for genetic effect size 0.5; *x*-axis, **A**,**B**), or over genes of different effect sizes (for 9 time points; *x*-axis **C**,**D**). Shown are different patterns of genetic effects (left to right: impulse, single state-transitioning (sustained), linear, and complex sub-panels). In **A**,**C**, shown is the accuracy measure, whereas in **B**,**D**, presented are the sensitivity and specificity measures (in solid and dashed lines, respectively; assuming *P* value 0.1), exemplified for DyVER against the PCA method (chosen since its accuracy is the best among the compared methods). Plots **A,C** indicate that for the non-linear genetic effect patterns, DyVER has an advantage in accuracy over existing approaches. The sensitivity and specificity tradeoff that leads to this accuracy advantage are demonstrated in **B** and **D**.
(EPS)

Figure S2 **Comparative performance analysis on nematode-based synthetic data.** Shown is accuracy (*y*-axis) for several compared approaches (color coded) using different patterns of genetic effects that were built based on the effect curves from Francesconi et al. (2014) (**Methods**). Results are shown over synthetic genes that were measured at six time points and of different genetic effect sizes (*x*-axis). The plot demonstrates that DyVER has an advantage over the compared approaches in a biological relevant synthetic data.
(EPS)

Figure S3 **DyVER's performance analysis using incomplete data.** The plot depicts the accuracy measures (*y*-axis) for the DyVER method across various percentages of missing data (0% [complete data], 20%, 40% and 60%, **Methods**) and for the compared methods in the case of complete data (*x-*axis). The results are shown for single state-transitioning (sustained) pattern of genetic effects, over genes that were measured at nine time points and genetic effect size 0.5. The complete data consists of the same 50 strains in each time points, where in the missing data input, each time point consists of a different (in some cases overlapping) list of strains (*e.g*., 30 selected strains in the 60% dataset). The plot indicates the high accuracy of DyVER, even in the case of missing data.
(EPS)

Figure S4 **Comparison of performance in a short-impulse and long-impulse synthetic data.** (**A**) Performance measures (*y*-axis) for different effect sizes (*x*-axis). The results presented are for all genes consisting of 27 time points with either short impulses (five time points, dashed lines) or long impulses (fifteen time points, solid lines). The plot depicts six alternative mapping methods (color coded). (**B**) The fraction of performance reduction for short impulses data compared to long impulses data (*y*-axis) for different effect sizes (*x*-axis). Results are shown for both accuracy (left) and sensitivity (right) measures, and are omitted when the accuracy or sensitivity in long-impulse data is low (<0.35 and <0.14, respectively). The plots indicate that fraction of performance reduction is much lower in the case of the DyVER algorithm than in the alternative methods, providing evidence for the good performance of DyVER in the case of short duration genetic effects.
(EPS)

Figure S5 **Effect pattern error rates.** Two-state pattern error rates using a stringent (**A**) or a flexible (**B**) matching (*y*-axis) for a model penalty ranging between 0.01 (high penalty) and 0.5 (no penalty; *x*-axis). Performances were evaluated for a single state-transitioning effect pattern dataset of nine time points. Results are shown for effect size 0.75 and using both stringent (red dashed line) and relaxed (red solid line) cutoff *P* values, as well as using random predictions (gray dashed line). As expected, in all cases, the higher the penalty, the lower the error rate.
(EPS)

Figure S6 **Non-linear genetic effect patterns.** Percentages of dynamically-associated genes predicted by DyVER (*y*-axis) across different non-linear genetic effect patterns (*x*-axis). Results are presented for analysis of real data (blue dots) and reshuffled data (box plots for 1000 repeats). Presented are results in yeast response to rapamycin (**A**, *cis*/top, *trans*/bottom), and in three-time-point data in mice strains (**B**, **Methods**). Notably, although the percentages are similar in yeast and mouse, their total number of genes drastically differ and therefore the actual number of identified genes is different (mouse – a total of 403 genes that were measured using the meso-scale nanostring nCounter technology; yeast – a total of 2700 top-ranking genes that were measured using the large-scale microarray technology).
(EPS)

Figure S7 **High correlations among genetic effects of consecutive time points.** (**A**) Presented is a correlation matrix of genetic effects between every pair of time points (red – high, blue - low). The matrix indicates that correlation among consecutive time points is higher than correlation among non-consecutive time points. (**B**) The distribution of mismatches between genetic effects of consecutive time points (t_i_ versus t_i+1_; red) and between genetic effects of non-consecutive time points (t_i_ versus t_i+2_; black). Depicted are three plot representing the fraction (*y* axis) of mismatch values (*x* axis) across all 105 non-linear dynamic associations (**Table S1**); top, middle and bottom panels represent t_i_  = 10, 20 and 30 minutes after rapamycin treatment, respectively. To calculate mismatch between genetic effects of two candidate time points, we first calculated a regression model relating genetic effects at a certain time point t_i_ (dependent variables) to genetic effects in the following time points t_i+1_ or t_i+2_ (independent variables). *Mismatch* values are defined as the residuals of this regression. The plots indicate that mismatches between consecutive time points are lower than mismatches between non-consecutive time points (Wilcoxon *P* values <<10^−15^ in all cases). (**C**) A heat map of the relations between genetic effects at 10 minutes (*y* axis), genetic effects at 20 minutes (*x* axis), and genetic effect at 30 minutes after rapamycin treatment (color coding; blue – low genetic effect; red – high genetic effect). Each cell represents a 2D bin consisting of all genes with genetic effects in a defined range at time points 10 and 20 minutes after treatment. 2D bins are colored based on their average genetic effect at 30 minutes after treatment (empty 2D bins are colored white). The heat map demonstrates that genetic effect at 30 minutes after treatment is linked to genetic effect in its nearby time point (20 min) but not to an earlier time point (10 min), consistently with DyVER's ‘memoryless’ Markov model.
(EPS)

Figure S8 **Non-mutually exclusive classes of temporal effect patterns in yeast and nematode.** Comparison between the fraction of genes (*y*-axis) that were classified into five non-mutually exclusive classes (*x*-axis) of the yeast dataset (Yeung et al., 2011; black) and the *C. elegance* dataset (Francesconi et al. (2014); white). Classification categories are as in Francesconi et al. (2014).
(EPS)

Figure S9 **Phosphate (Pi) acquisition and storage module no 3.** (**A**) Module no. 3 genes (pink) in the context of the phosphate acquisition and storage pathway (adapted from Ref. 29). The pathway shows the extracellular conversion of phosphate monoester into phosphate, phosphate transport into the cytoplasm, and deposition of phosphate into storage vacuoles. (**B**) The genomic interval underlying module no. 3, residing in Chr13: 28 kb. Shown are DyVER scores (*y-*axis) across the genomic positions in chromosome 13 (*x*-axis) for the genes in module no. 3 (color coded). The position of the known causal variants in *PHO84* is marked below; all remaining genes are *trans*-associated. Genetic effects of the module and a representative gene are depicted in **Fig. 5B–E** **.** (**C**) Genetic effects, relative to non-stimulated genetic effects (*y*-axis, log scaled) for different *trans*-associated genes from **B** (color coded) at six time points (*x*-axis).
(EPS)

Figure S10 **Genetic effects and transcription responses of co-associated genes in module no. 4.** (**A**) Genetic effects, relative to non-stimulated genetic effects (*y*-axis, log scaled) for the co-associated genes in module no. 4 (color coded) at six time points (*x*-axis). (**B**) Averaged transcription response, relative to non-stimulated transcription response (*y*-axis, log scaled) for module no. 4 genes (color coded) at six time points (*x*-axis). Notably, the module genes share a similar genetic effect pattern (**A**), even though they do not share a similar transcription response pattern (**B**).
(EPS)

Figure S11 **Identifying the genes likely underlying the nitrogen-regulated module no. 5 - II.** (**A**) Potential causal genes underlying the nitrogen-regulated module no. 5-II (column 1), genetic linkage interval at chromosome 2 (column 2). Amino acid differences between the RM and BY strains are reported in column 3. The table presents three selection criteria: First, by reporting genes whose temporal transcription profiles fit the expected impulse effect pattern (column 4, detailed in **B,C**). Second, by reporting those genes that are significantly bound by nitrogen-related transcription factors (column 5, detailed in **D**). Finally, by reporting functionally-related genes (that is, genes known to be involved in nitrogen or amino acid pathways, column 6). Shown are all genes selected by at least one criterion. Notably, *RPB5* and *CNS1* were selected by all three criteria (marked in **Fig. 6A**). (**B**) Averaged temporal gene expression profiles (blue color scale) of the genes in the linkage interval of module no. 5-II (rows) following rapamycin stimulation (columns). Genes showing a specific high expression level at ten minutes following stimulation are marked in arrows and listed in **A** (*RPB5*, *CNS1*). Plot (**C**) demonstrates the agreement between the averaged transcription response of *RPB5* and *CNS1* (solid lines) and the averaged relative genetic effect pattern in module no. 5-II (dashed line, *y*-axis) during time points (*x*-axis). (**D**) Shown is a –log *P* value of transcription factor binding data (from Ref. 32, *y*-axis) for the genes in the linkage interval of module no. 5-II (white, *x*-axis). The two transcription factors, *DAL80* (top) and *GCN4* (bottom) are known as key regulators of the nitrogen and amino acid pathways. A threshold corresponding to the level of binding in known nitrogen-related genes (black) is indicated in dashed horizontal line. Genes with a similar or higher binding –log *P* value are listed in **A**.
(EPS)

Figure S12 **DyVER's predicted two-state model for dynamic genes in yeast following rapamycin treatment.** Shown is a table of cluster identifiers (column 1) and their number of genes (column 2). The partition was generated automatically according to DyVER's predicated two-state model. The model for each cluster is shown either as a sequence of 'L' and 'H' states (column 3) or in a cartoon visualization (column 4; ‘H’ - light blue, ‘L’ – white). The pattern in columns 3 and 4 is shown for increasing time points from left to right. For example, the LLLHHH pattern indicates a high genetic effect only at 30–50 minutes after rapamycin treatment.
(EPS)

Figure S13 **Q-Q plots for the DyVER's score.** (**A**) An example of two QQ-plots of representative genes. The plots show no inflation and deflation of the expected minus log *P* values (*x-*axis) versus the observed minus log *P* values of the DyVER score (*y-*axis). Genomic control (GC) values were defined as the median of the observed minus log *P* value divided by the median of the minus log expected *P* value. (**B**) An overall distribution (box-plot) of GC values across all genes in the dataset. As expected, the distribution of genomic control values is centered in genomic control  = 1. Plots **A** and **B** were generated using a synthetic dataset of 500 genes that were measured at nine time points using single state-transitioning (sustained) pattern with genetic effect size  = 0.5.
(EPS)

Figure S14
**Three possible formulations of the DyVER's likelihood ratio test.** (**A**) A table presenting the three formulations (rows); including the name of the approach (column 1), its likelihood ratio formulation and parameters (column 2) and the degrees of freedom that should be used for a χ^2^− approximation of *P* values (column 3). In all cases, the null hypothesis is an absence of an effect and the alternative hypothesis is the presence of an effect. The formulation of the DyVER score is specified in line no. 1. The additional formulations I and II (in lines 2 and 3, respectively) are focused only on the 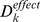 dataset: Assuming the presence of an effect, the full two-state model is used as in the DyVER score. Assuming the absence of an effect, the mean value of both the high-effect and low-effect states is set to zero; formulation I assumes a different variance for the low-effect and high-effect states, whereas formulation II assumes an equal variance. (**B**,**C**) Comparative performance analysis on synthetic data. Scatter plots for the accuracy measure (*y*-axis) of different methods (color coded), including (i) the five existing approaches (implemented as detailed in **Methods**) and (ii) three formulations of the DyVER's likelihood ratio tests as specified in **A** (*P* values for all three methods were derived using a permutation test). Results are shown over synthetic genes with a single state-transitioning (sustained) pattern of genetic effects; the genes were measured in different numbers of time points (*x*-axis, **B**) or different effect sizes (*x*-axis, **C**), as presented in **Fig. 3**. The plots clearly show that all three formulations of the DyVER's likelihood ratio test provide similar performance.(EPS)

Figure S15 **Performance analysis of the PCA approach on synthetic data.** Scatter plots for the accuracy measure (*y*-axis) of three possible PCA-based methods over synthetic genes with a single state-transitioning (sustained) pattern of genetic effects; the genes were measured in different numbers of time points (*x*-axis) for genetic effect size 0.5. Different line types indicate the results for PC1, PC2 and PC3, respectively. The plot demonstrates that the accuracy attained by the first component is the best among the consecutive components.
(EPS)

Table S1 **DyVER's predicted associated genes in yeast following rapamycin treatment.** Shown are 145 gene symbols (column 1), their genomic position (column 2), the genomic position of their associated genetic variant (column 3) and whether it is associated in *cis* or in *trans* (column 4). Column 5 provides information about the predicted two-state model of the association (L - low-effect state, H - high effect state). The timeline (0-50 minutes) is ordered from left to right. For example, the LLLHHH pattern indicates a high genetic effect only at 30–50 minutes after rapamycin treatment.
(DOC)

Table S2 **A comparison between previously reported genetic variants and DyVER's predictions.** The table presents genomic position of all previously reported genetic variants (column 1), their known causal gene (column 2) and the particular condition in which the genetic variant was identified (column 3). DyVER's predictions are presented in columns 4–5: Column 4 provides the non-linear genes significantly associated with the variant (based on the DyVER score; *cis* associations are highlighted in bold), whereas columns 5 indicates the corresponding module number as listed in **Fig. 5A**. ^*^non-significant DyVER score. References for known causal genes: ^1^Smith and Kryglyak. 2008, ^2^Perlstein et al. 2007 ^3^Brem et al. 2005, ^4^Yvert et al. 2003, ^5^Brem et al. 2002 and Gaisne et al. 1999.
(DOC)
