# Supplementary material for: Dissecting Dynamic Genetic Variation That Controls Temporal Gene Response in Yeast
Source: PLoS Comput Biol. 2014 Dec 4;10(12):e1003984. doi: 10.1371/journal.pcbi.1003984 (PMC4256076; doi:10.1371/journal.pcbi.1003984)
Supplement: Table S1 — DyVER's predicted associated genes in yeast following rapamycin treatment. Shown are 145 gene symbols (column 1), their genomic position (column 2), the genomic position of their associated genetic variant (column 3) and whether it is associated in cis or in trans (column 4). Column 5 provides information about the predicted two-state model of the association (L - low-effect state, H - high effect state). The timeline (0-50 minutes) is ordered from left to right. For example, the LLLHHH pattern indicates a high genetic effect only at 30–50 minutes after rapamycin treatment. (DOC) [file pcbi.1003984.s016.doc]

**Table S1**

**Table S1 – cont.**

**Table S1 – cont.**
